# X-ray-Induced Changes in the Expression of Inflammation-Related Genes in Human Peripheral Blood

**DOI:** 10.3390/ijms151119516

**Published:** 2014-10-27

**Authors:** Ping Wang, Fei Guo, Lin Han, Xi’ai Wang, Jie Li, Yan Guo, Yumin LÜ

**Affiliations:** 1 Department of Toxicology, Henan Institute of Occupational Medicine, Zhengzhou 450052, China; E-Mails: wangping980623@126.com (P.W.); zhifangsuohanlin@126.com (L.H.); zhzhwxa@126.com (X.W.); lijie1101@sina.cn (J.L.); 2 College of Public Health, Zhengzhou University, Zhengzhou 450001, China; E-Mails: guofei_19870427@163.com (F.G.); guoyan.208@163.com (Y.G.)

**Keywords:** X-ray, human peripheral blood, inflammation-related genes, quantitative PCR array

## Abstract

Using quantitative real-time polymerase chain reaction (PCR) array, we explored and compared the expression changes of inflammation-related genes in human peripheral blood irradiated with 0.5, 3, and 10 Gy doses of X-rays 24 h after exposure. Results indicated that the expression of 62 out of 84 genes was significantly altered after X-ray radiation. Among these 62 genes, 35 (such as *TNFSF4*) are known to be associated with radiation response, but others are novel. At a low radiation dose (0.5 Gy), 9 genes were up-regulated and 19 were down-regulated. With further increased dose to 3 Gy, 8 unique genes were up-regulated and 19 genes were down-regulated. We also identified 48 different genes that were differentially expressed significantly after 10 Gy of irradiation, and among these transcripts, up-regulated genes accounted for only one-third (16 genes) of the total. Of the 62 genes, 31 were significantly altered only at a specific dose, and a total of 10 genes were significantly expressed at all 3 doses. The dose- and time-dependent expression of *CCL2* was confirmed by quantitative real-time reverse-transcription PCR. A number of candidate genes reported herein may be useful molecular biomarkers of radiation exposure in human peripheral blood.

## 1. Introduction

Exposure to ionizing radiation—whether for medical, occupational, or accidental reasons—may cause deleterious biological consequences, such as mortality or carcinogenesis. No dose of ionizing radiation exposure is considered safe, and the accurate estimation of the absorbed dose is critical in determining appropriate medical care. Hence, in the event of a large-scale radiation accident or attack, radiation dosimetry assays are imperative to assess the amount of radiation exposure as quickly as possible to accurately triage victims for appropriate care. The metaphase chromosomal aberration assay in human lymphocytes remains as the gold standard for assessing radiation dosimetry, and no functional equivalent has been found. However, this assay is labor intensive, requires mitotic cells, lacks specificity at very low and high doses, and requires skilled individuals [1,2]. Therefore, developing additional radiation dosimetry assays that are high throughput, automated and cost efficient is imperative [3]. Over the past few decades, numerous efforts have been exerted to design a fast, reliable, effective, easy, and cost-effective biological dosimeter that requires minimum invasive procedures. Currently, ionizing radiation-induced RNA-based gene expression change is the focus of a new generation of research on radiation biodosimetry [4]. RNA-based assays mainly include global expression platforms such as DNA microarray and expression assessment by quantitative real-time reverse-transcription polymerase chain reaction (qRT-PCR). DNA microarrays can reportedly be used to simultaneously identify and quantify thousands of ionizing radiation-induced expression changes within a single sample [5,6,7]. However, sweeping research methods, numerous verification works, and the complexities of objective things seriously limit the application of DNA microarrays in the area of radiobiology, particularly in biodosimetry. Quantitative real-time polymerase chain reaction (qPCR) array, which screens differentially expressed genes based on mRNA, is a highly sensitive and reliable method of gene-expression profiling. qPCR array combines qRT-PCR and gene chips and allows the simultaneous quantitative monitoring of expression levels of panel genes in a specific pathway or disease-related genes. qPCR array is a useful tool for functional genomic studies [8,9] and may thus be an ideal method for screening early biodosimetry. With the development of functional genomics research, qPCR array is becoming critical in studies on life science topics, such as drug resistance of tumors [10], pathogenic microorganisms [11,12], cellular and developmental biology [13,14], cytokines and inflammatory reactions [15,16], specific biological pathways [17], *etc.* However, studies on the applications of qPCR array in the field of radiobiology, especially biodosimetry, are few.

In the last decade, accumulated data from several researchers have indicated that low-dose radiation therapy (single dose = 0.5–1 Gy) can functionally modulate a wide multitude of inflammatory processes and cellular compounds [18]. With the help of microarray or qPCR, thousands of scholars have identified numerous inflammation-related genes associated with radiation-induced expression changes, such as *IL1A*, *IL1B*, *IL6*, *BLC*, *C10*, *IP-10*, *CCL2*, *CCL7*, *MIP-1γ*, *RANTES*, *CCR1*, *CCR2*, *CCR5*, *CCR6*, *etc.* [19,20]. The expression changes of these genes have been identified after a single dose irradiation; only one study has screened out a small part of inflammation-related genes. However, most inflammation-related genes simultaneously induced after low-, medium- and high-dose exposures have not been reported.

In the present work, we examined changes in the expression of inflammation-related genes in peripheral blood before and after exposure to a single dose (0, 0.5, 3, and 10 Gy) at the mRNA expression level of DNA damage response. We also determined whether the dose- or time-dependent expression of *CCL2* was significant. The purpose was to identify inflammation-related genes with radiation-induced expression changes in human peripheral blood cells after *in vitro* irradiation. Another purpose was to better understand gene expression changes associated with low-, medium-, and high-dose radiation, which may provide insight into the molecular mechanisms of inflammatory reactions induced by ionizing radiation and lead to the development of novel biodosimeters.

## 2. Result and Discussion

### 2.1. Comparison of Transcriptional Profiles of Human Peripheral Blood Cells after Exposure to Different X-ray Doses

**Figure 1:**
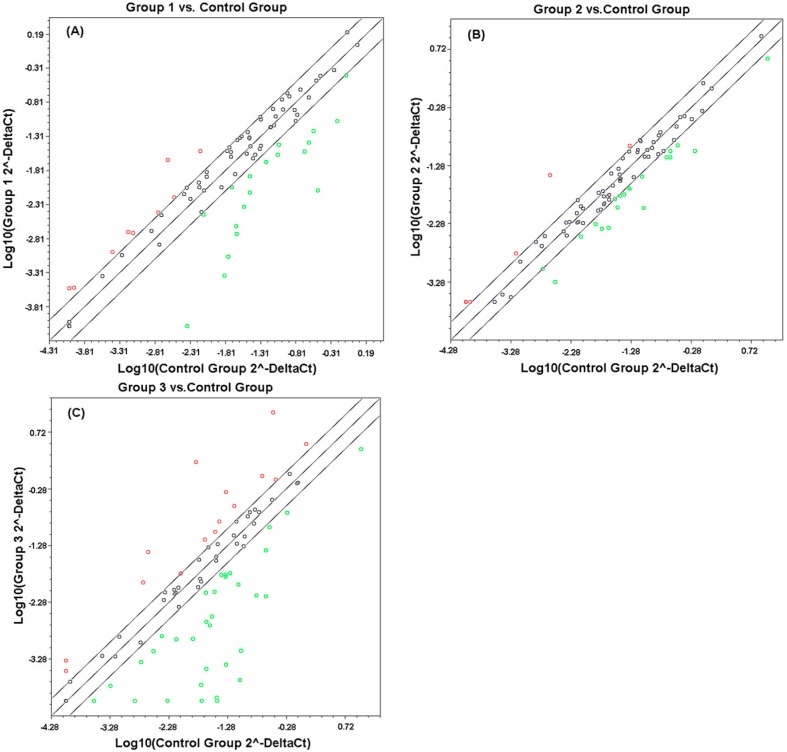
Scatter plot analysis representative of transcriptional profiles of human peripheral blood cells 24 h after exposure to different X-ray doses ((**A**–**C**) 0.5, 3, and 10 Gy, respectively). This figure shows genes with increased (red color) or decreased (green color) expression after radiation compared with those of sham-irradiated cells; Genes with |fold regulation| < 2 were indicated in black.

X-ray-induced gene expression changes were examined in a homogeneous population of human peripheral blood cells obtained from two normal donors (a 34-year-old male and a 32-year-old female). Two independent qPCR array analyses were carried out for each radiation dose after the *in vitro* reverse transcription reaction of RNA extracted from human peripheral blood cells. These cells were harvested 24 h after 0.5, 3, and 10.0 Gy of X-ray exposure or sham irradiation. Genes modulated at least twofold in each individual qPCR array experiment were considered reliable radiation-responsive genes. Based on analysis criteria, the number of radiation-inducible genes ranged from 27–48 genes, *i.e.*, ~28.1%–50.0% of the total genes in the qPCR array, in terms of the dose tested (Table 1). Up-regulated and down-regulated genes were almost equally distributed in the 0.5, 3 and 10 Gy sets. Figure 1 shows a scatter plot analysis representative of genes with altered expression. Clearly, a certain set of genes was induced by a specific dose or dose range. A total of 48 genes showed altered expression induced by 10 Gy. This number of X-ray-inducible genes was the largest among all investigated doses, thereby confirming that exposures to high doses more dramatically affected gene expression than low doses. The genes with altered expression at 0.5, 3, and 10 Gy are listed in Table 2.

**Table 1:** Numbers of genes with changed transcription levels in irradiated human peripheral blood cells.

Expression Changes	Gene Number		
	0.5 Gy	3 Gy	10 Gy
Up-regulated	9	8	16
Down-regulated	19	19	32
Total	28	27	48

**Figure 2:**
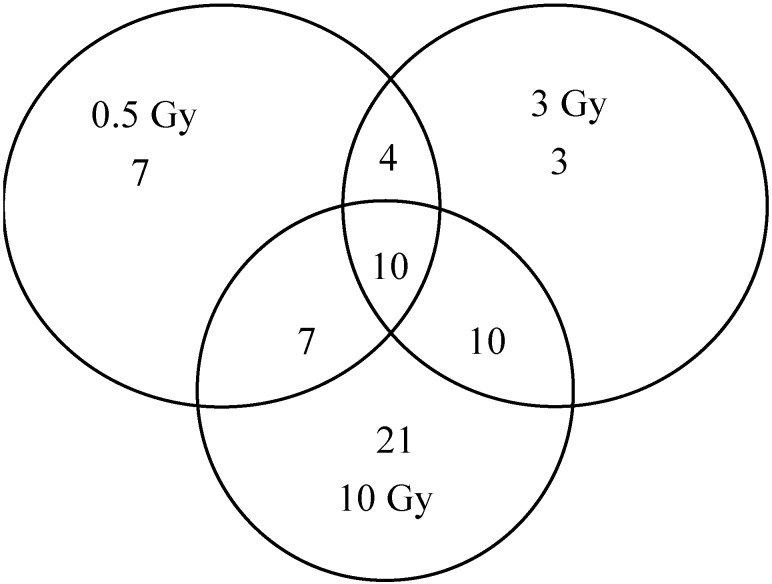
Venn diagram showing the overlap of genes with significant expression changes 24 h after exposure to different X-ray doses.

Global analysis of qPCR array data indicated that compared with the control, the mRNA levels of 62 genes (Table 2 and Figure 2) exhibited significant expression changes after exposure to 0.5, 3, or 10 Gy of X-ray radiation. Conversely, insignificant expression changes were observed in the expression of 22 genes, including *BCL2*, *BIRC2*, *BIRC3*, *CASP8*, *CCL5*, *CFLAR*, *CHUK*, *CTSB*, *HSP90AB1*, *IKBKB*, *MAPK1*, *MAPK8*, *NAIP*, *NOD1*, *NFKB1*, *NLRP9*, *PYCARD*, *RELA*, *TAB1*, *TAB2*, *TRAF6*, and *XIAP* at all doses (data not shown). At the transcriptional level, a considerable overlap was observed at 24 h in response to low-, medium-, and high-dose exposures (Figure 2). Among these 62 genes, 14 responded to the low and medium doses, 17 genes were significantly affected after both low and high doses, 20 genes exhibited significant expression differences at both medium and high doses, and 10 genes (including *CCL2*, *MEFV*, *CXCL1*, *CXCL2*, *P2RX7*, *PTGS2*, *TNF*, *NLRX1*, *IFNB1*, and *TNFSF4*) were common to all doses tested. For example, IFNB1 and TNFSF4 were up-regulated, but some other genes (including *MEFV*, *CXCL2*, *P2RX7*, *PTGS2*, and *TNF*) were all down-regulated at all doses. Moreover, *TNFSF4* mRNA expression was up-regulated with increased irradiation dose, in contrast to *PTGS2* mRNA expression. Compared with the control, one gene (*NLRX1*) was up-regulated in low dose but down-regulated in medium and high doses, but two genes (*CCL2* and *CXCL1*) were down-regulated in low and medium doses but up-regulated in a high dose 24 h after X-ray radiation. Furthermore, some genes were up-regulated at low and medium doses but down-regulated at a high dose.

**Table 2:** Profiling of genes associated with X-ray-induced inflammatory reaction.

Symbol	GenBank	Description	Fold Regulation		
			0.5 Gy	3 Gy	10 Gy
*CCL2*	NM_002982	Chemokine (C–C motif) ligand 2	−39.5871	−2.6739	37.1193
*CXCL1*	NM_001511	Chemokine (C–X–C motif) ligand 1 (melanoma growth stimulating activity, alpha)	−7.0153	−2.8083	4.2787
*NLRX1*	NM_024618	NLR family member X1	2.1804	−5.4679	−4.0013
*MEFV*	NM_000243	Mediterranean fever	−6.3207	−2.0551	−9.6336
*CXCL2*	NM_002089	Chemokine (C–X–C motif) ligand 2	−5.9388	−2.5035	−35.3923
*P2RX7*	NM_002562	Purinergic receptor P2X, ligand-gated ion channel, 7	−9.4334	−4.9114	−2.8395
*PTGS2*	NM_000963	Prostaglandin-endoperoxide synthase 2 (prostaglandin G/H synthase and cyclooxygenase)	−3.2288	−8.6517	−382.4951
*TNF*	NM_000594	Tumor necrosis factor	−4.7188	−2.1824	−359.0338
*IFNB1*	NM_002176	Interferon, beta 1, fibroblast	3.0783	2.5595	3.4564
*TNFSF4*	NM_003326	Tumor necrosis factor (ligand) superfamily, member 4	9.3354	15.2667	17.2687
*IL1B*	NM_000576	Interleukin 1, beta	−7.3595	−6.4265	
*IL6*	NM_000600	Interleukin 6 (interferon, beta 2)	−20.1839	−3.9760	
*NLRP4*	NM_134444	NLR family, pyrin domain containing 4	2.6747	2.1798	
*TNFSF11*	NM_003701	Tumor necrosis factor (ligand) superfamily, member 11	2.9932	2.5231	
*CASP5*	NM_004347	Caspase 5, apoptosis-related cysteine peptidase	−12.5221		−63.2637
*CCL7*	NM_006273	Chemokine (C–C motif) ligand 7	−33.7867		103.5741
*IRF2*	NM_002199	Interferon regulatory factor 2	−2.8683		−3.4050
*MAPK13*	NM_002754	Mitogen-activated protein kinase 13	−2.7257		−313.9804
*NLRP1*	NM_033004	NLR family, pyrin domain containing 1	−2.3960		−119.3437
*NLRP3*	NM_183395	NLR family, pyrin domain containing 3	−2.1744		−197.9183
*PEA15*	NM_003768	Phosphoprotein enriched in astrocytes 15	−4.6696		−2.4521
*CARD18*	NM_021571	Caspase recruitment domain family, member 18		2.5595	5.3355
*SUGT1*	NM_006704	SGT1, suppressor of G2 allele of SKP1 ( *S. cerevisiae*)		2.3183	9.3633
*CIITA*	NM_000246	Class II, major histocompatibility complex, transactivator		−2.6392	−11.2611
*IKBKG*	NM_003639	Inhibitor of kappa light polypeptide gene enhancer in B-cells, kinase gamma		−2.2396	−2.9962
*IRAK1*	NM_001569	Interleukin-1 receptor-associated kinase 1		−3.1312	−3.8438
*IRF1*	NM_002198	Interferon regulatory factor 1		−3.2151	−5.4590
*MAPK3*	NM_002746	Mitogen-activated protein kinase 3		−2.3435	−119.1749
*NFKBIB*	NM_002503	Nuclear factor of kappa light polypeptide gene enhancer in B-cells inhibitor, beta		−2.4474	−2.5170
*NLRC5*	NM_032206	NLR family, CARD domain containing 5		−2.3274	−7.4314
*NOD1*	NM_006092	Nucleotide-binding oligomerization domain containing 1		−2.0327	−3.8188
*CD40LG*	NM_000074	CD40 ligand	4.3937		
*IL12A*	NM_000882	Interleukin 12A (natural killer cell stimulatory factor 1, cytotoxic lymphocyte maturation factor 1, p35)	2.5043		
*MAPK12*	NM_002969	Mitogen-activated protein kinase 12	2.5661		
*MOK*	NM_014226	Renal tumor antigen	2.1817		
*IL12B*	NM_002187	Interleukin 12B (natural killer cell stimulatory factor 2, cytotoxic lymphocyte maturation factor 2, p40)	−55.6021		
*IL18*	NM_001562	Interleukin 18 (interferon-gamma-inducing factor)	−2.2094		
*NFKBIA*	NM_020529	Nuclear factor of kappa light polypeptide gene enhancer in B-cells inhibitor, alpha	−2.0706		
*IL33*	NM_033439	Interleukin 33		2.5595	
*NLRP5*	NM_153447	NLR family, pyrin domain containing 5		2.5595	
*BCL2L1*	NM_138578	Bcl2-like 1		−2.4431	
*AIM2*	NM_004833	Absent in melanoma 2			2.0252
*HSP90AA1*	NM_001017963	Heat shock protein 90 kDa alpha (cytosolic), class A member 1			2.2279
*HSP90B1*	NM_003299	Heat shock protein 90 kDa beta (Grp94), member 1			2.8132
*IFNG*	NM_000619	Interferon, gamma			6.1451
*MAP3K7*	NM_003188	Mitogen-activated protein kinase kinase kinase 7			2.8662
*MYD88*	NM_002468	Myeloid differentiation primary response gene (88)			3.8568
*NLRC4*	NM_021209	NLR family, CARD domain containing 4			3.6631
*PANX1*	NM_015368	Pannexin 1			2.0607
*RIPK2*	NM_003821	Receptor-interacting serine-threonine kinase 2			3.1423
*CARD6*	NM_032587	Caspase recruitment domain family, member 6			−5.9438
*CASP1*	NM_033292	Caspase 1, apoptosis-related cysteine peptidase (interleukin 1, beta, convertase)			−23.2608
*FADD*	NM_003824	Fas (TNFRSF6)-associated via death domain			−100.3419
*MAPK11*	NM_002751	Mitogen-activated protein kinase 11			−2.9619
*MAPK9*	NM_002752	Mitogen-activated protein kinase 9			−12.8590
*NLRP12*	NM_033297	NLR family, pyrin domain containing 12			−14.3709
*NLRP6*	NM_138329	NLR family, pyrin domain containing 6			−9.6825
*PSTPIP1*	NM_003978	Proline-serine-threonine phosphatase-interacting protein 1			−3.3141
*PYDC1*	NM_152901	PYD (pyrin domain) containing 1			−2.9089
*TIRAP*	NM_001039661	Toll-interleukin 1 receptor (TIR) domain containing adaptor protein			−3.0328
*TNFSF14*	NM_003807	Tumor necrosis factor (ligand) superfamily, member 14			−51.3506
*TXNIP*	NM_006472	Thioredoxin-interacting protein			−2.7110

Among the 62 genes, 31 were altered only at a specific dose. Among genes that responded only to a low dose, 4 (*CD40LG*, *IL12A*, *MAPK12*, and *MOK*) were up-regulated and 3 (*IL12B*, *IL18*, and *NFKBIA*) were down-regulated; these were induced only after a medium dose radiation. There were only 3 genes, including the up-regulation of 2 genes (*IL33* and *NLRP5*), and the down-regulation of 1 gene (*BCL2L1*) that exhibited only significant expression changes following exposure to the highest dose; only 42.9% (9 genes, including *AIM2*, *HSP90AA1*, *HSP90B1*, *IFNG*, *MAP3K7*, *MYD88*, *NLRC4*, *PANX1*, and *RIPK2*) were up-regulated, whereas 57.1% (12 genes, including *CARD6*, *CASP1*, *FADD*, *MAPK11*, *MAPK9*, *NLRP12*, *NLRP6*, *PSTPIP1*, *PYDC1*, *TIRAP*, *TNFSF14*, and *TXNIP*) were down-regulated.

**Figure 3:**
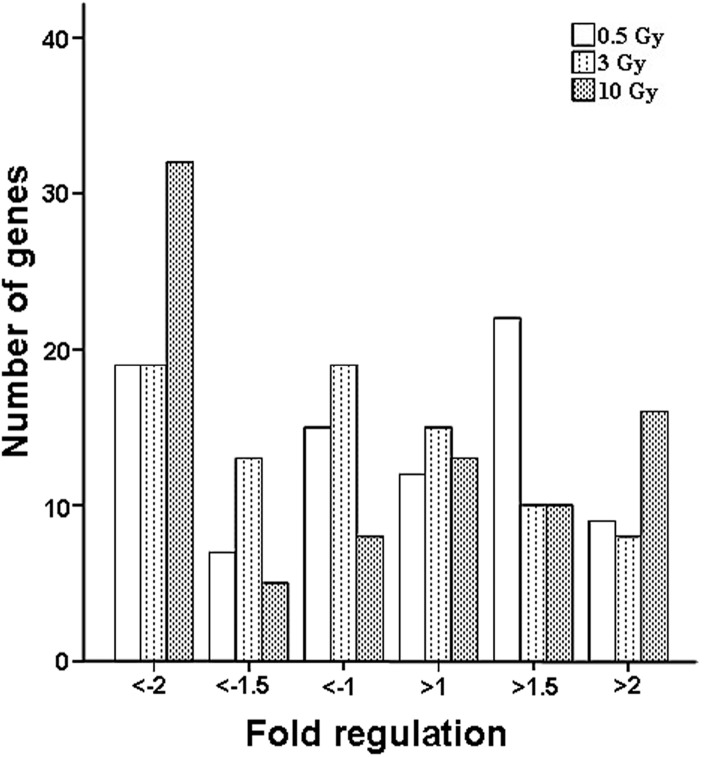
Distribution of fold regulations of all genes tested after a low-, medium-, or high-dose exposure.

We also assessed the scale of variation of all genes tested on the qPCR array at low, medium, and high doses. Apart from genes with significant differential expression, fold changes of the majority of the tested genes were between 1.5 and 2 (Figure 3). We observed few genes showing significant alteration in expression at all doses at the twofold threshold. However, when the threshold was reduced to 1.8-fold, some genes including *BIRC2*, *CTSB*, *MAPK1*, *NLRP9*, *PYCARD*, and *TAB2* were also found to have significant differential expression. Moreover, *PEA15*, *NLRP4*, and *PSTPIP1* were affected by all doses tested. Fold changes of 2.0 [21,22,23] or 1.5 [24,25] are common cutoff values in microarray experiments. Thus, when the threshold was reduced 1.5-fold, *BCL2*, *BIRC3*, *CFLAR*, *NFKB1*, and *TAB1* were considered as having significant differential expression. Seventeen genes (including *BIRC2*, *CASP5*, *CCL7*, *CHUK*, *CIITA*, *IFNG*, *IL12A*, *IL1B*, *IL6*, *IRF2*, *MAPK11*, *MAPK8*, *NFKBIB*, *NLRC4*, *NLRP3*, *PANX1*, and *SUGT1*) with significantly changed expression were common to all irradiation doses. Moreover, 4 genes (*IFNG*, *IL12A*, *MAPK8* and *SUGT1*) were found to be persistently up-regulated, 4 genes (*CASP5*, *CIITA*, *NFKBIB*, and *NLRP3*) were persistently down-regulated, and the remaining genes were up-regulated or down-regulated after a single dose of 0.5, 3, or 10 Gy of irradiation.

### 2.2. Comparison of qPCR Array with Other Studies

Using high-throughput gene chips or cDNA microarrays, thousands of scholars have identified a large number of genes. These genes play important roles in signal transduction, cell cycle regulation, cytoskeleton organization and cell motility, DNA repair, and apoptosis in response to ionizing radiation [26,27,28,29,30]. However, only a small part of these genes affect the inflammation process. In the present work, we used qPCR array to screen out 62 genes with significant differential expression (|fold regulation| > 2) associated with inflammatory reaction after X-ray irradiation. Including *BCL2L1*, *CARD6*, *CASP1*, *CASP5*, *CCL2*, *CCL7*, *CD40LG*, *CXCL1*, *CXCL2*, *FADD*, *HSP90AA1*, *HSP90B1*, *IFNB1*, *IFNG*, *IKBKG*, *IL12A*, *IL12B*, *IL18*, *IL1B*, *IL6*, *IRAK1*, *IRF1*, *MAP3K7*, *MAPK12*, *MAPK3*, *MYD88*, *NFKBIA*, *NLRP12*, *NLRP3*, *PTGS2*, *SUGT1*, *TNF*, *TNFSF11*, *TNFSF14*, and *TNFSF4*, 35 were associated with ionizing radiation response, as previously reported in different types of cells, tissues, and organisms [31,32,33,34,35,36,37,38,39,40,41,42,43,44,45,46,47,48,49,50,51,52,53,54,55,56]. Among these genes, 27 (including *AIM2*, *CARD18*, *CIITA*, *IL33*, *IRF2*, *MAPK11*, *MAPK13*, *MAPK9*, *MEFV*, *NFKBIB*, *NLRC4*, *NLRC5*, *NLRP1*, *NLRP4*, *NLRP5*, *NLRP6*, *NLRX1*, *NOD2*, *P2RX7*, *PANX1*, *PEA15*, *PSTPIP1*, *PYDC1*, *MOK*, *RIPK2*, *TIRAP*, and *TXNIP*) have not previously been shown to be responsive to ionizing radiation damage at the mRNA level (|fold regulation| > 2). Ten genes responded to all doses (0.5, 3, and 10 Gy), and only *NLRX1*, *MEFV*, and *P2RX7* were novel. Among 84 genes, 22 did not show any significant change in expression (the primers of these 22 genes were included in RT^2^ Profiler PCR Arrays in 96-well plates). However, previous studies have shown that some of these genes, namely, *BCL2*, *BIRC2*, *BIRC3*, *CASP8*, *CCL5*, *CFLAR*, *CHUK*, *HSP90AB1*, *IKBKB*, *MAPK8*, *NAIP*, *NOD1*, *NFKB1*, *PYCARD*, *RELA*, *TRAF6*, and *XIAP*, exhibited significant expression changes at the mRNA level after irradiation [31,32,33,37,45,49,50,51,52,57,58,59,60,61]. Variations in radiation type, dose rate, tissue type, and cell types used in the different studies may account for the differences in results. Moreover, fold regulations of *BCL2*, *BIRC2*, *BIRC3*, *CASP8*, *CCL5*, *CFLAR*, *CHUK*, *CTSB*, *MAPK1*, *MAPK8*, *NFKB1*, *NLRP9*, *PYCARD*, *TAB1*, *TAB2*, and *TRAF6* were more than 1.5 or less than −1.5 after exposure. If the cutoff values were reduced 1.5-fold, the above genes can also be considered as having significantly differential expression, but only *CTSB*, *MAPK1*, *NLRP9*, *TAB1*, and *TAB2* were novel. However, fold regulations of *HSP90AB1*, *IKBKB*, *NAIP*, *NOD1*, *RELA*, and *XIAP* were less than 1.5 or more than −1.5 after exposure; thus, they still cannot be considered to have significant differential expression even when the cutoff values were reduced 1.5-fold. However, these genes have been previously shown to be responsive to ionizing radiation damage at the mRNA level [31,32,37,50,51,52].

We subsequently tested the ability of the 62-gene signature to predict the exposure doses of individual samples under blinded conditions. Expression information for the 62 genes was used to build a Nearest Centroid Classifier [62,63] with leave-one-out cross-validation to predict samples as belonging to unexposed, 0.5, 3, or 10 Gy categories. The dose and time dependence of inflammation-related genes (except *TNFSF4*), which were closely associated with ionizing radiation response after irradiation remained unclear. Pogosova-Agadjanyan *et al.* [64] reported that *TNFSF4* expression in CD3+ lymphocytes, mononuclear cells (MNCs), and white blood cell fraction (MNC+ granulocytes) exhibited significant linear dose-dependent expression changes 24 h after 2, 6, and 12 Gy of radiation (γ-rays; 7.6 Gy/min). Results of the qPCR arrays suggested that fold changes of *TNFSF4* mRNA-altered expression were 9.3354, 15.2667, and 17.2687 after 0.5, 3, and 10 Gy of irradiation (X-ray; 100 cGy/min), respectively. This means that the expression changes of *TNFSF4* mRNA were up-regulated with increased irradiation dose. We also found that expression changes of *TNFSF4* mRNA exhibited a dose-dependent response within a wide range of doses (0, 1, 3, 5, 8, 10, and 12 Gy) that was independent of post-irradiation incubation time. Moreover, expression was not affected by age and gender in 51 healthy donors [65]. Thus, *TNFSF4* is a possible candidate gene of ionizing radiation that can be used to assess radiation exposure and toxicity. As for others, especially those common to all the irradiation doses or the medium and high dose, we can further study the dose-dependent or time-dependent response. If some genes exhibit a dose-dependent response, we can account them into a model which will be developed to estimate *in vitro* radiation exposures using a stepwise regression procedure (SAS PROC REG) [64]. Ideally, candidate genes selected for building biodosimeter models exhibit certain dose-dependent expression changes unassociated with age, gender, time, cell type, and/or other potential inter-individual confounders after irradiation. Radiation dosimetry assays using such target genes could be implemented and interpreted across a wide variety of human populations with potentially infinite exposure scenarios [64]. Therefore, further study on the dependence of these genes on age, gender, cell type, or other potential inter-individual confounders is necessary.

### 2.3. Validation of X-ray-Induced Expression Changes of CCL2 in Human Peripheral Blood

**Figure 4:**
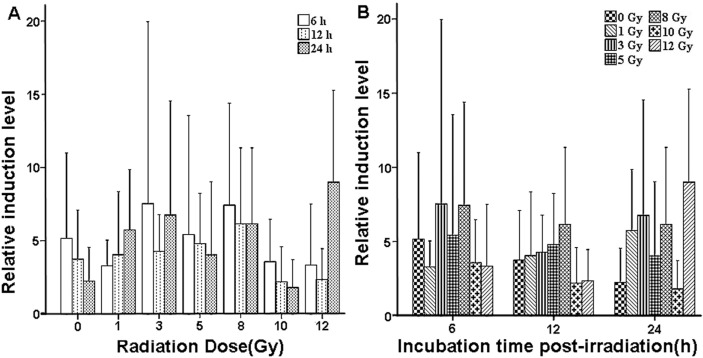
Expression of *CCL2* in human peripheral blood after X-ray irradiation. (**A**) Dose-dependent expression change; and (**B**) time-dependent expression change. Error bars represent standard deviation (*n* = 7 donors).

The expression changes of *CCL2*, which was observed to be down-regulated at low and medium doses but up-regulated at a high dose 24 h after X-ray radiation in qPCR array, were validated by qRT-PCR. These changes were then examined in a homogeneous population of human peripheral blood cells obtained from seven healthy volunteer donors (age range = 23–33; four males and three females). The qRT-PCR assay results are shown in Table 3 and Figure 4. No significant statistical difference was found among the different doses (F = 0.920, *p* = 0.495; Figure 4A). The differences among incubation time post-irradiation were also not statistically significant (*F* = 0.190, *p* = 0.783; Figure 4B). Thus, *CCL2* had radiation-induced expression levels that were not highly dependent on the X-ray dose exposure or incubation time post-irradiation. However, the expression levels of *CCL2* mRNA exhibited a certain trend of up-regulation within a wide range of doses 24 h after irradiation.

**Table 3:** X-ray-induced expression changes of *CCL2* mRNA in human peripheral blood.

Dose (Gy)	Relative Induction Level Relative Induction		
	6 h	12 h	24 h
0	5.1547 ± 5.8280	3.7394 ± 3.3493	2.2400 ± 2.3159
1	3.1912 ± 1.7462	4.0470 ± 4.3098	5.7423 ± 4.1118
3	7.5279 ± 12.4202	4.2638 ± 2.5147	6.7529 ± 7.7754
5	5.4198 ± 8.1194	4.8103 ± 3.4379	4.0393 ± 4.9922
8	7.4343 ± 6.9659	6.1453 ± 5.1910	6.1574 ± 5.1804
10	3.5730 ± 2.9049	2.2096 ± 2.3899	1.8030 ± 1.9142
12	3.3331 ± 4.1677	2.3643 ± 2.0885	8.9944 ± 6.2734

CCL2 (chemokine (C–C motif) ligand 2), also known as HC11, MCAF, MCP1, MCP-1, SCYA2, GDCF-2, SMC–CF, or HSMCR30, is an important member of the C–C subfamily of chemokines. These chemokines are produced by some tumor cells and macrophages and characterized by having two adjacent cysteine residues. CCL2 plays important roles in attracting MNCs to inflammatory sites and in regulating macrophage functions. The expression of monocyte chemotactic protein-1/CCL2 may reportedly be an independent prognostic marker for gastric cancer [66] and a link between hepatic inflammation, fibrosis and, angiogenesis [67]. Four-month-old Fisher 344-Brown Norway (F344 × BN) male rats received whole brain irradiation with a single dose of 10 Gy γ-rays (^137^Cs; 4.23 Gy/min). A significant and marked up-regulation of mRNA and protein expression of monocyte chemoattractant protein-1 (MCP-1) was observed in hippocampal and cortical regions isolated from irradiated brain 4, 8, and 24 h after irradiation [68]. A single dose of 8 Gy to the brain of 14-day-old postnatal mice had caused up-regulation of CCL2 mRNA expression level 6 h after irradiation [69]. After exposing Male Wistar rats to single dose-irradiation (25 Gy; 2.4 Gy/min), CC–Chemokine (*MCP-1*/*CCL2* and *MCP-3*/*CCL7*) gene expression had been significantly induced in total RNA from irradiated livers 3–6 h after irradiation [34]. In [52], 23-day-old C57BL/6 mice (male and female) have been exposed to 2, 10 Gy, or fractionated ionizing radiation (2 Gy per day for 5 days (2 Gy × 5)) (γ-rays; 0.81 Gy/min). Results show that *CCL2* mRNA expression is significantly (more than twofold) up-regulated in non-targeted heart after 2 or 10 Gy but not after 2 Gy × 5. In [20], the thoraces of 8-week-old female mice of C57BL/6 (fibrosis sensitive) strain have been irradiated with a single dose of 12.5 Gy at a dose rate of about 3 Gy/min using a ^137^Cs γ-ray source. The expression of *CCL2* and *CCL7* mRNA are found to be elevated in irradiated mice compared with age-matched controls 26 weeks after irradiation. In some other studies, it was found that *CCL2* mRNA was up-regulated in tumor cell lines (SCC-4, SCF-7, IOMM Lee, SF-3061, or A549) receiving X-ray or γ-ray irradiation [50,51,70,71]. All these previous results indicate that the expression level of *CCL2* significantly increases regardless of *in vitro* or *in vivo* exposure. In the present study, qRT-PCR results 24 h after irradiation (except at 10 Gy dose) agreed with the aforementioned previous results. Differences in irradiation source, dose rate, time and manner of irradiation, donor characteristic, culture condition, cell, tissue, or organ may account for the discrepancies in the results of the present and previous studies.

qPCR array analysis showed that fold changes of altered expression of *CCL2* mRNA were −39.5871, −2.6739, and 37.1193, 24 h after 0.5, 3, and 10 Gy of irradiation (X-rays; dose rate = 100 cGy/min), respectively. In other words, *CCL2* was up-regulated after high-dose irradiation (10 Gy). This finding was consistent with other previous studies but different from the present qRT-PCR results. Analysis of qPCR array indicated that *CCL2* was down-regulated at low and medium doses (0.5 and 3 Gy), which disagreed with qRT-PCR results possibly because of differences in housekeeping gene, primer design, donor characteristic, or volume of added medium.

Though *CCL2* was not the most significant among these genes identified in this study, we wondered whether *CCL2* exhibits significant linear dose-dependent expression changes within the wider dosage range and can be a potential candidate gene for biological dosimeters. Thus, we simultaneously examined expression changes of *CCL2* and *TNFSF4* [65] at different times after varied irradiation doses. In addition to *TNFSF4* and *CCL2*, we next also need to test other radiation-inducible genes affected by all doses tested in qPCR array assay, especially *PTGS2*. Further study is needed for both dose- and time-dependent responses, as well as their distribution in normal populations.

## 3. Materials and Methods

### 3.1. Acquisition of Samples from Healthy Donors and Ethics Statement

Peripheral blood samples were collected from healthy volunteer donors (*n* = 2 for qPCR array analysis; *n* = 7 for qRT-PCR assay) at the Henan Institute of Occupational Medicine (HIOM). The scope and risk of the study were explained to each subject before written informed consents were obtained. The Medical Ethics Committee of HIOM (Zhengzhou, China) approved all experiments in this study (2012-05, 16 July 2012). All subjects had no history of chronic disease, substance abuse, toxic chemical exposure, radiation exposure, or viral infection during the months preceding the study.

### 3.2. In Vitro Irradiation of qPCR Array Analysis Samples and RNA Extraction

Whole blood samples from each of two healthy donors (one male and one female) were irradiated with X-ray at room temperature using MEVATRON medical electron linear accelerator (The First Affiliated Hospital of Zhengzhou University, Zhengzhou, China) at an average rate of 100 cGy/min with doses of 0.5, 3 and 10 Gy. The uniform exposure field was 40 cm × 40 cm. After irradiation, the cells were maintained in a 37 °C constant-temperature incubator and cultured in 20 mL of RPMI-1640 medium (HyClone, Logan, UT, USA) containing 10% heat-inactivated fetal bovine serum (SunBao Biotech, Shanghai, China) and 160 U/mL gentamicin. The cells were then harvested at 24 h to prepare RNA extracts. RNA was extracted using BQIAamp RNA Blood Mini Kit (Qiagen, Hilden, Germany) according to the manufacturer’s recommendations and assessed for the quality and concentration of each sample with NanoVueTM (GE Healthcare, Princeton, NJ, USA). Spectrophotometrical measurements showed that all samples had (i) *A*_260_:*A*_230_ ratio above 1.7; (ii) *A*_260_:*A*_280_ ratio within 1.8–2.0; and (iii) concentration determined by *A*_260_ more than 40 μg/mL.

### 3.3. Inflammation-Related Gene Expression Measurement Using qPCR Array

First, 500 ng of total RNA was reverse transcribed into cDNA using RT^2^ First Strand Kit (Qiagen) following the manufacturer’s protocol. Second, 500 ng of cDNA equivalent was used for each profiling plate mixed with RT^2^ SYBR Green Mastermix (Qiagen) on an ABI 7500 RT-PCR System (Applied Biosystems, Foster City, CA, USA) following the manufacturer’s instructions. Data were evaluated using Sequence Detection Software 1.3.1 (Applied Biosystems) combined with Excel^®^ (Microsoft, Unterschleissheim, Germany) for statistical evaluation. RT^2^ Profiler PCR Arrays (Qiagen) in 96-well plates used in the study contained primer assays for 84 inflammation-related genes and 5 housekeeping genes (*ACTB*, *B2M*, *GAPDH*, *HPRT1* and *RPLP0*). One well contained a genomic DNA control, 3 wells contained reverse-transcription controls, and 3 wells contained positive PCR controls. We started with a highly selective qPCR profiler rather than a global gene array because the selected genes in qPCR array had a well-characterized profile governing inflammatory signal transduction and transcriptional targets. Thus, data interpretation, simplification of data acquisition, data analysis, and gene function determination can be facilitated. qPCR profiling also allows real-time detection and quantification of gene expression. ΔΔ*C*_t_ values were calculated by normalizing the gene expression levels to those of 5 housekeeping genes and then comparing with controls. The relative expression level of each gene was expressed as fold regulation. When comparing each gene’s signal intensity between irradiated and sham-irradiated groups, we set a cutoff of ≥two-fold for gene induction and ≤two-fold for gene repression.

### 3.4. In Vitro Irradiation of qRT-PCR Assay Samples and Subsequent Cell Isolation

Whole blood samples from seven healthy volunteer donors were irradiated at 0 (sham), 1, 3, 5, 8, 10, and 12 Gy as described above. After irradiation, the cells were cultured in 12 mL of RPMI-1640 medium (HyClone, Logan, UT, USA) as described above and then harvested at 6, 12, and 24 h. White blood cells were separated as previously described [72].

### 3.5. Chemokine (C–C Motif) Ligand 2 (CCL2) Expression Measurement Using qRT-PCR

RNA was extracted from white blood cells using Trizol reagent (Invitrogen, Carlsbad, CA, USA) per the manufacturer’s recommendations and assessed for quality and concentration as described above. Spectrophotometrical measurements showed that all samples had (i) *A*_260_:*A*_280_ ratio within 1.8–2.0 and (ii) concentration determined by *A*_260_ more than 57.1 μg/mL. For all qRT-PCR studies, 1000 ng of total RNA was reverse transcribed with random primer using reverse transcription M-MMLV (Takara, Dalian, China) according to the manufacturer’s guidelines. Each reverse transcription product was diluted to 40 μL with sterilized double-distilled water. Then, 50 ng of cDNA equivalent was then assayed on an ABI 7500 RT-PCR system (Applied Biosystems) mixed with the components of SYBR^®^Premix Ex TaqTM (Takara, Dalian, China) according to the manufacturer’s instructions. The forward (*GAPDH*: 5'-GGAGAAGGCTGGGGCTCAT-3'; *CCL2*: 5'-CTCATAGCAGCCACCTTCATTC-3') and reverse (*GAPDH*: 5'-TGGGTGGCAGTGATGGCA-3'; *CCL2*: 5'-GAGTGTTCAAGTCTTCGGAGTTTG-3') primers were PCR primer sequences for qRT-PCR designed according to MITOMAP Human Cambridge Sequence data [73] and then synthesized by Gene Core Bio Technologies Co., Ltd., Shanghai, China. Fluorescence signals were normalized by the internal control dye (ROX II). ΔΔ*C*_t_ values were calculated by normalizing the gene expression level to the endogenous control glyceraldehyde 3-phosphate dehydrogenase 1 (GAPDH). All samples were run in triplicates with appropriate blank controls. Data are presented as the mean ± SD and analyzed using IBM SPSS software Version 21.0 (SPSS, Chicago, IL, USA). *p* values <0.05 were considered statistically significant. All reported *p* values were two-sided. Repeated measurement ANOVA was used to determine the statistical significance of irradiated blood samples.

## 4. Conclusions

We identified 62 significant differentially expressed genes related to inflammation following X-ray irradiation using qPCR array, such as *IFNB1*, *TNFSF4*, *CCL2*, and *CCL7*. We found that the qPCR array technique was a powerful tool for screening radiation-responsive genes and can be used to explore the molecular mechanism of inflammation as influenced by genes that were differentially expressed significantly under ionizing radiation. We also examined dose- and time-dependent responses of CCL2 after X-ray irradiation. The results can help in the development of early, rapid, and high-throughput biological dosimeters.
